# [^99m^Tc]Tc-iFAP Radioligand for SPECT/CT Imaging of the Tumor Microenvironment: Kinetics, Radiation Dosimetry, and Imaging in Patients

**DOI:** 10.3390/ph15050590

**Published:** 2022-05-11

**Authors:** Luis Coria-Domínguez, Paola Vallejo-Armenta, Myrna Luna-Gutiérrez, Blanca Ocampo-García, Brenda Gibbens-Bandala, Francisco García-Pérez, Gerardo Ramírez-Nava, Clara Santos-Cuevas, Guillermina Ferro-Flores

**Affiliations:** 1 Department of Radioactive Materials, Instituto Nacional de Investigaciones Nucleares, Ocoyoacac 52750, Mexico; angel.c.servicios@inin.gob.mx (L.C.-D.); paovallejoarmenta@gmail.com (P.V.-A.); myrna.luna@inin.gob.mx (M.L.-G.); blanca.ocampo@inin.gob.mx (B.O.-G.); brenda.g.servicios@inin.gob.mx (B.G.-B.); 2 Faculty of Medicine, Universidad Autónoma del Estado de México, Toluca 50180, Mexico; 3 Department of Nuclear Medicine, Instituto Nacional de Cancerología, Tlalpan, Mexico City 14080, Mexico; fosvaldogarcia@gmail.com

**Keywords:** fibroblast activation protein, FAP imaging, technetium-99m, [^99m^Tc]Tc-FAP inhibitor ligand

## Abstract

Tumor microenvironment fibroblasts overexpress the fibroblast activation protein (FAP). We recently reported the preclinical evaluation of [^99m^Tc]Tc-iFAP as a new SPECT radioligand capable of detecting FAP. This research aimed to evaluate the kinetic and dosimetric profile of [^99m^Tc]Tc-iFAP in healthy volunteers, and to assess the radioligand uptake by different solid tumors in three cancer patients. [^99m^Tc]Tc-iFAP was obtained from lyophilized formulations prepared under GMP conditions with >98% radiochemical purity. Whole-body scans of six healthy subjects were obtained at 0.5, 2, 4, and 24 h after [^99m^Tc]Tc-iFAP (740 MBq) administration. A 2D-planar/3D-SPECT hybrid activity quantitation method was used to fit the biokinetic models of the source organs (volume of interest: *VOI*) as exponential functions (*A(t)_VOI_*). The total nuclear transformations (*N*) that occurred in the source organs were calculated from the mathematical integration (*0,∞*) of *A(t)_VOI_*. The OLINDA code was used to estimate the radiation doses. Three treatment-naive patients (breast, lung, and cervical cancer) with a prior [^18^F]FDG PET/CT scan underwent whole-body, chest, and abdominal SPECT/CT scanning after [^99m^Tc]Tc-iFAP (740 MBq) administration. Both imaging methods were compared visually and quantitatively. Oncological diagnoses were performed histopathologically. The results showed favorable [^99m^Tc]Tc-iFAP biodistribution and kinetics due to rapid blood activity removal (t_1/2_α = 2.22 min and t_1/2_β = 90 min) and mainly renal clearance. The mean radiation equivalent doses were 5.2 ± 0.8 mSv for the kidney and 1.7 ± 0.3 mSv for the liver after administration of 740 MBq. The effective dose was 2.3 ± 0.4 mSv/740 MBq. [^99m^Tc]Tc-iFAP demonstrated high and reliable uptake in the primary tumor lesions and lymph node metastases in patients with breast, cervical, and lung cancer, which correlated with that detected by [^18^F]FDG PET/CT. The tumor microenvironment molecular imaging from cancer patients obtained in this research validates the performance of additional clinical studies to determine the utility of [^99m^Tc]Tc-iFAP in the diagnosis and prognosis of different types of solid tumors.

## 1. Introduction

Fibroblasts are ubiquitously present throughout the body and exhibit high dipeptidyl peptidase-4 (serine exopeptidase enzyme) expression but usually no expression of the fibroblast activation protein (FAP), a serine endopeptidase/exopeptidase enzyme. In contrast, cancer-associated fibroblasts (CAFs) express FAP, which cleave proteins at any post-proline bond in the amino acid sequence [1,2].

The tumor microenvironment is the setting around a tumor, including surrounding blood vessels, signaling molecules (e.g., growth factors, chemokines, and cytokines), the extracellular matrix, immune cells, and CAFs [3]. Therefore, FAP is specifically expressed in the tumor microenvironment of more than 90% of epithelial tumors, especially in carcinomas characterized by a significant desmoplastic reaction (breast, colon, and pancreatic cancer), which contributes to cancer progression and poor disease prognosis [1].

The N-(4-quinolinoyl)-glycyl-(2-cyano-4,4-difluoropyrrolidine) molecule is an effective FAP inhibitor (FAPI) [4,5,6]. Based on this structure, a dozen radiolabeled FAPI derivatives, most of them for PET (positron emission tomography) imaging, have been reported for cancer diagnosis [4,5,6,7,8,9,10,11,12,13]. For example, [^68^Ga]Ga-FAPI-04 PET/CT imaging of 28 different tumors was successfully obtained from 80 patients [7]. In fact, [^68^Ga]Ga-FAPI-04 has demonstrated potential as a broad-spectrum tumor imaging agent, except for multiple myeloma and lymphoma [14]. There have also been reports concerning the superiority of [^68^Ga]Ga-FAPI-04 when compared to [^18^F]FDG PET/CT in the detection of primary and metastatic tumors in hepatocarcinoma and colorectal, duodenal, and gastric cancers [14,15,16,17,18].

[^99m^Tc]Tc-FAPI-35, a N-(4-quinolinoyl)-glycyl-(2-cyano-4,4-difluoropyrrolidine) radiotracer, has been successfully used in cancer detection of pancreatic and ovarian cancer via SPECT (single-photon emission computed tomography) [11].

Considering that, worldwide, there is a higher number of SPECT cameras compared to PET, our group reported the synthesis and preclinical evaluation of the [^99m^Tc]Tc-((R)-1-((6-hydrazinylnicotinoyl)-D-alanyl)pyrrolidin-2-yl) boronic acid ([^99m^Tc]Tc-iFAP) as a novel FAP inhibitor radiotracer for SPECT imaging of the tumor microenvironment [19]. [^99m^Tc]Tc-iFAP boronic acid showed stability in human serum, specific recognition of FAP, fast kidney elimination, and high tumor uptake (7.05% ID/g) [19].

This research aimed to evaluate the kinetic and dosimetric profile of [^99m^Tc]Tc-iFAP in healthy volunteers, and to assess radioligand uptake by different solid tumors in three patients.

## 2. Results

### 2.1. Biokinetics and Dosimetry

**Figure 1:**
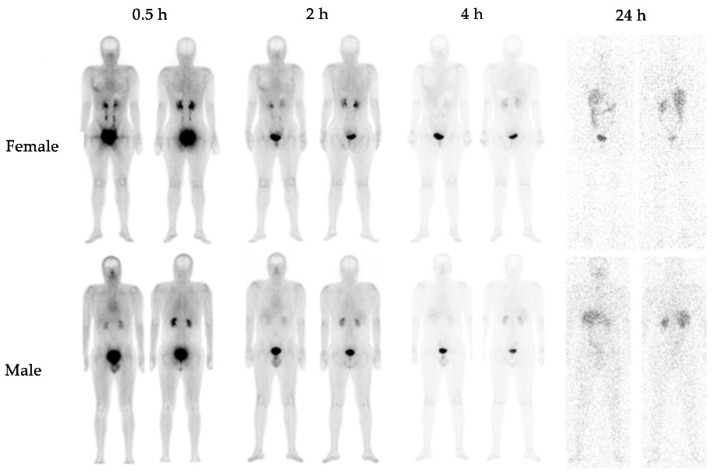
Anterior and posterior planar whole-body images of healthy subjects (**top**: female, **bottom**: male) at 0.5 h, 2 h, 4 h, and 24 h after [^99m^Tc]Tc-iFAP intravenous administration (740 MBq).

No adverse events related to the diagnostic use of [^99m^Tc]Tc-iFAP were observed in patients and healthy subjects. The physiological biodistribution of the radioligand in the whole-body scan was observed in cardiac cavities (pool), the kidney, and the liver to a lesser extent, with biliodigestive and renal elimination (Figure 1). Radiotracer uptake was outlined around the female mammary gland mainly associated with tissue blood flow, which was scarce after 4 h.

**Figure 2:**
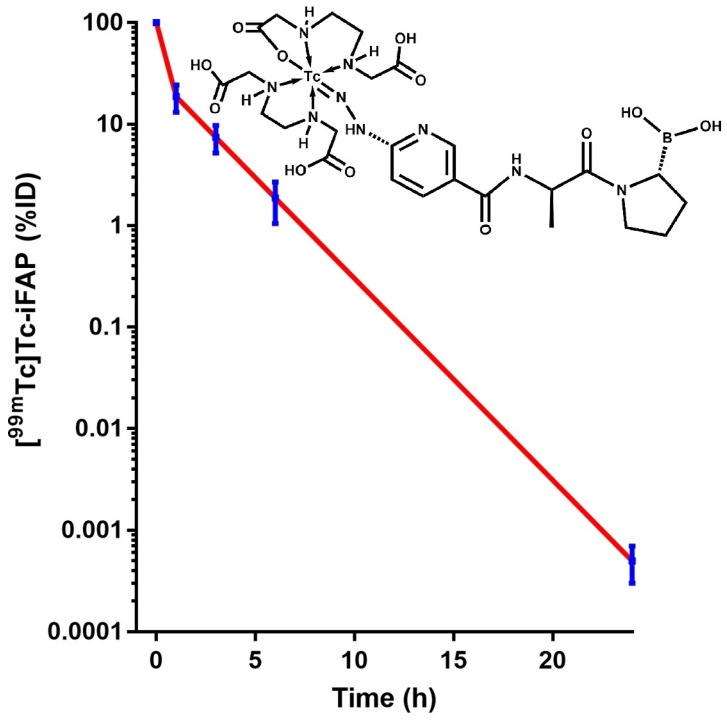
Blood clearance of [^99m^Tc]Tc-iFAP from healthy subjects with t_1/2_α = 2.22 min (70.4%) and t_1/2_β = 90 min (29.6%). Inset: proposed chemical structure for the [^99m^Tc]Tc-iFAP radioligand.

The [^99m^Tc]Tc-iFAP kinetic model in the blood indicated that the clearance of activity fits a two-component exponential function (Equation (1)) (Figure 2). The first component showed that 70.4% of the radiotracer was removed from the blood with a half-life of 2.22 min (t_1/2_α = ln 2/18.52 = 0.037 h), and 29.6% (second component) was cleared from the blood with a half-life of 90 min (t_1/2_β= ln 2/0.46 = 1.5 h) (Equation (1)). One hour after radioligand injection, the percentage of the dose in the kidneys was 7.69 ± 4.80%, and it decreased to 0.36 ± 0.14% at 24 h. As expected, the highest number of total nuclear transformations occurred in the urinary bladder followed by the kidney and the liver (Table 1).

(1) $$A{\left(t\right)}_{Blood\;}=70.40e^{-18.52t}+29.60e^{-0.46t}\;R^{2}=1$$

The radiation doses of [^99m^Tc]Tc-iFAP are shown in Table 2. The kidney is the organ that received the highest equivalent dose. For an activity of 740 MBq, the kidney equivalent dose received by the six healthy subjects was 5.2 ± 0.8 mSv, and 1.7 ± 0.3 mSv was found for the liver, with an effective dose of 2.3 ± 0.4 mSv.

For low effective doses (from 2 mSv to 50 mSv), the radiation risk can be correlated with the natural background radiation. Therefore, the dose received from [^99m^Tc]Tc-iFAP (2.3 mSv) is equal to that received over 8 months from the natural background radiation. The calculated effective dose for [^99m^Tc]Tc-iFAP is comparable with the doses for most radiopharmaceuticals at around <10 mSv [20], which indicates a minimal risk in agreement with the United Nations Scientific Committee on the Effects of Atomic Radiation (UNSCEAR) [21,22].

### 2.2. [^99m^Tc]Tc-iFAP Tumor Imaging in Patients

Table 3 describes the clinical characteristics of the three patients included for both [^99m^Tc]Tc-iFAP and [^18^F]FDG tumor imaging.

**Figure 3:**
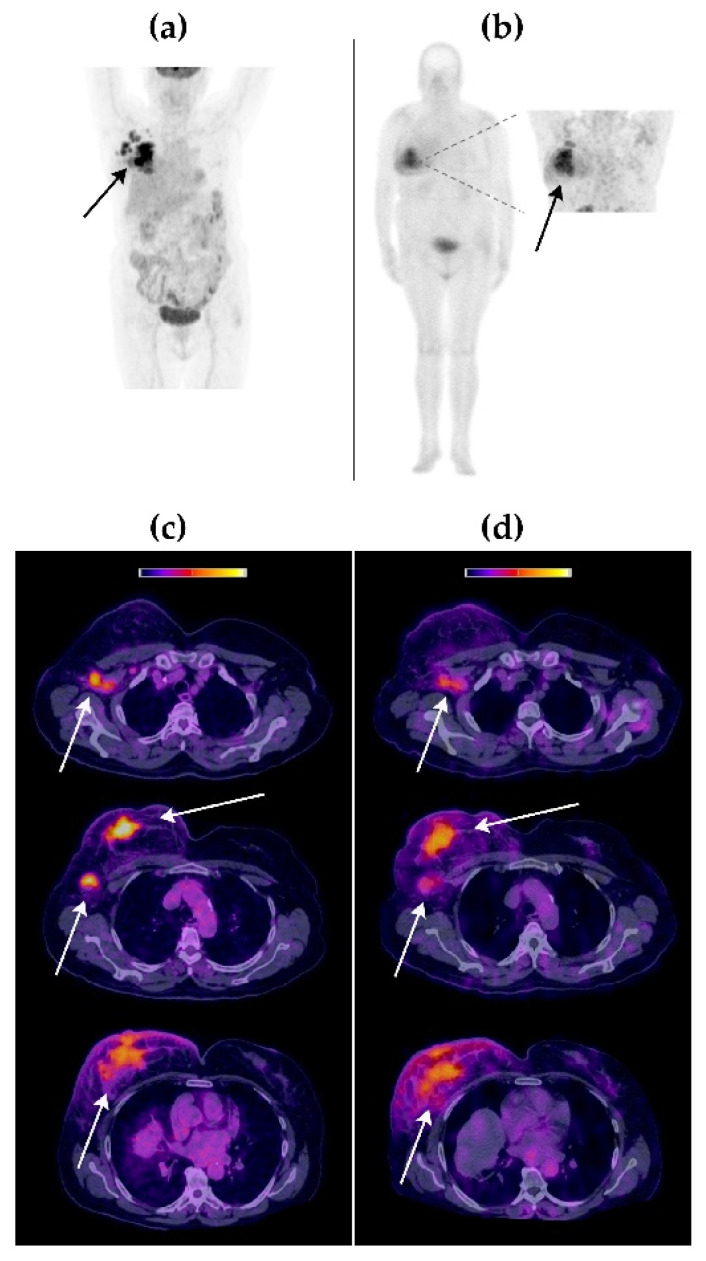
Patient with triple-negative right breast cancer (Patient 1). (**a**) Maximum-intensity projection [^18^F]FDG PET image at 1 h p.i. (**b**) [^99m^Tc]Tc-iFAP whole-body planar scintigraphy in anterior projection. Inset: thoracic SPECT at 3 h p.i. (**c**) [^18^F]FDG PET/CT axial sections. (**d**) [^99m^Tc]Tc-iFAP SPECT/CT axial sections. Right mammary gland with increased size and thickening of the generalized cutaneous plane, with a solid lesion as well as poorly defined borders in the interline topography of the upper quadrants that extends to the retroareolar region, conditioning its retraction (primary tumor). Ipsilateral axillary lymphadenopathies (Berg levels I and II). The uptake of both radiopharmaceuticals was concordant in all tumor lesions.

**Figure 4:**
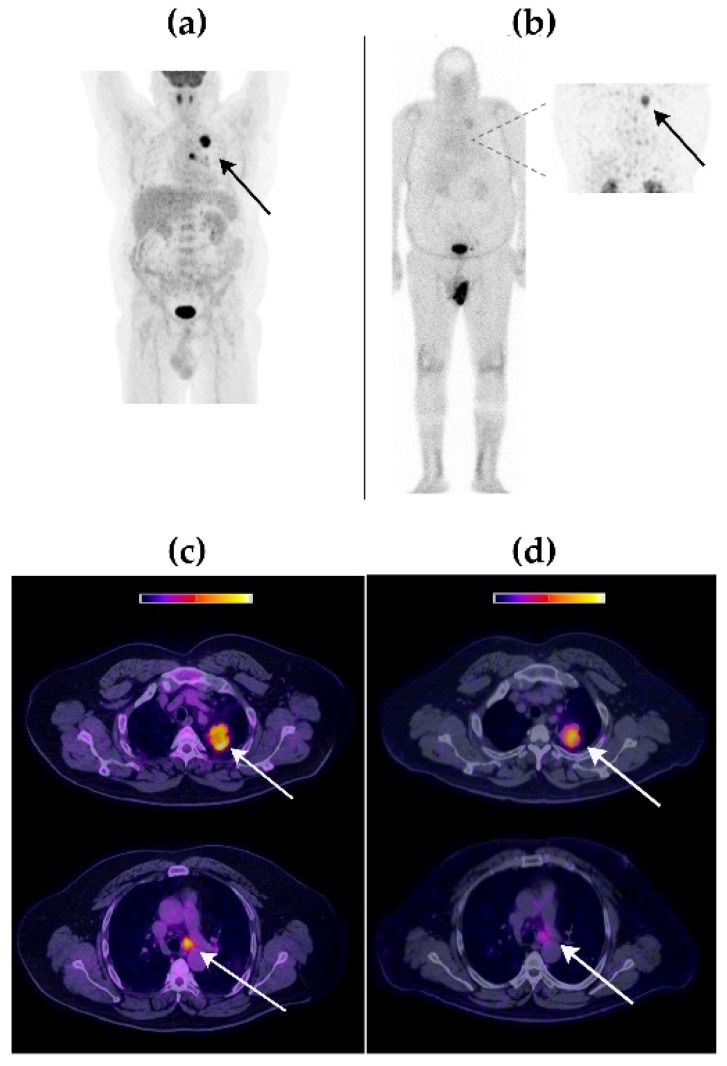
Patient with left lung adenocarcinoma (Patient 2). (**a**) Maximum-intensity projection [^18^F]FDG PET image at 1 h p.i. (**b**) [^99m^Tc]Tc-iFAP whole-body planar scintigraphy in anterior projection. Inset: thoracic SPECT at 3 h p.i. (**c**) [^18^F]FDG PET/CT axial sections. (**d**) [^99m^Tc]Tc-iFAP SPECT/CT axial sections. Solid lesion in the apicoposterior segment of the left upper lobe with irregular edges, which contacts the mediastinal pleura. Mediastinal adenopathy at subaortic level 5. The uptake of both radiopharmaceuticals was concordant in all tumor lesions.

**Figure 5:**
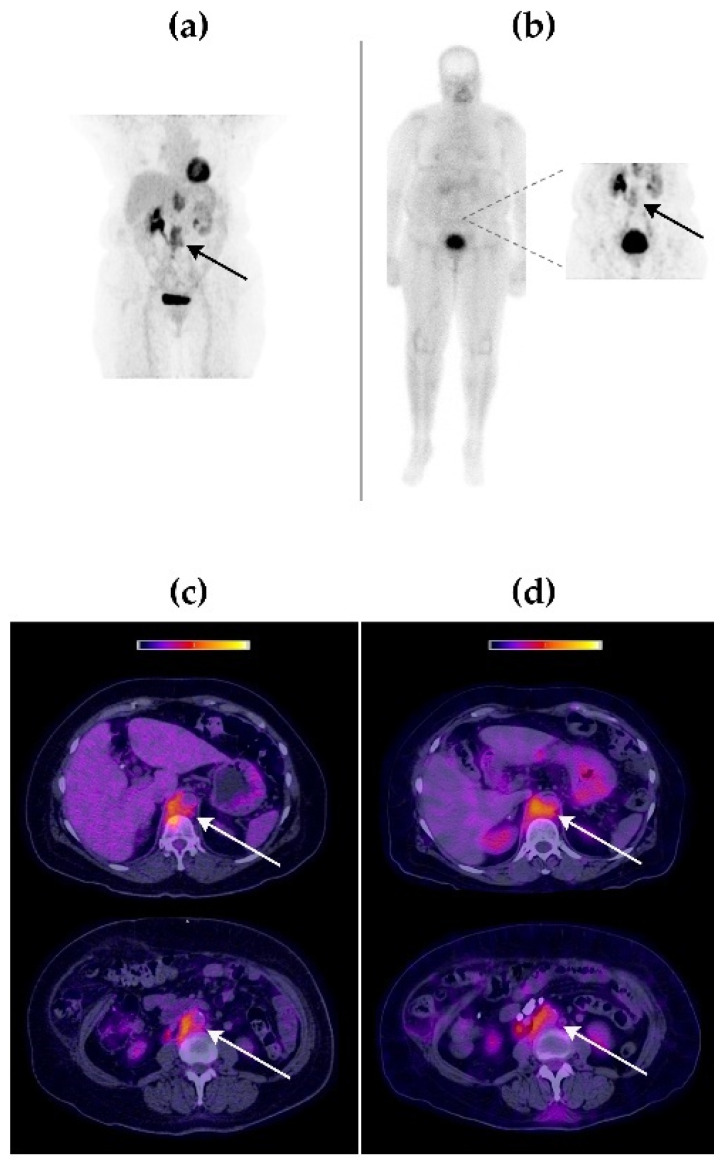
Patient with cervical squamous cell carcinoma and retroperitoneal lymph node recurrence (Patient 3). (**a**) Maximum-intensity projection [^18^F]FDG PET image at 1 h p.i. (**b**) [^99m^Tc]Tc-iFAP whole-body planar scintigraphy in anterior projection. Inset: abdominal SPECT at 3 h p.i. (**c**) [^18^F]FDG PET/CT axial sections. (**d**) [^99m^Tc]Tc-iFAP SPECT/CT axial sections. Lymph node conglomerates at the right retrocrural and retroperitoneal intercaval-aortic level with retrocaval extension. No recurrent primary lesion was observed at the pelvic level. The uptake of both radiopharmaceuticals was concordant in all tumor lesions.

Visual comparison between [^99m^Tc]Tc-iFAP SPECT/CT and [^18^F]FDG PET/CT molecular imaging techniques revealed that both radiotracers accumulated in the same tumor lesions, with an excellent image quality (Figure 3, Figure 4 and Figure 5). 

The [^99m^Tc]Tc-iFAP radiotracer concentration was higher in cervical lesions (40.4 kBq/cm^3^) than in breast (22.4 kBq/cm^3^) and lung (17.6 kBq/cm^3^) tumors. In the case of [^18^F]FDG, the highest SUVmax was obtained in lung lesions (15.3), followed by those in the breast (SUVmax of 12.5) and cervix (SUVmax of 10.7). Tumor lesions were divided into primary tumors and lymph node metastases (Table 4). Table 4 also shows the tumor/background (T/B) ratios calculated from [^99m^Tc]Tc-iFAP SPECT/CT and [^18^F]FDG PET/CT patients’ images. The results of the three-way ANOVA analysis with Tukey’s multiple comparisons test show that there was no statistically significant difference (*p* > 0.05) regarding T/B ratios between [^99m^Tc]Tc-iFAP and [^18^F]FDG. However, there was a significant difference (*p* < 0.05) in the T/B ratios among the different types of cancer and between primary tumors and lymph node metastases. It is important to note that the T/B ratios of [^18^F]FDG, especially muscle uptake (T/Bp), are variable and depend on external factors, such as fasting and physical activity, which does not occur with [^99m^Tc]Tc iFAP. This factor can be an advantage for obtaining homogeneous and reproducible T/B ratios, regardless of the previous preparation of the patient. In agreement with other reports and our previous preclinical studies [5,6,11,18], a rapid and high tumor uptake of [^99m^Tc]Tc iFAP was observed, as well as tumor efflux. Nevertheless, tumors were visualized with excellent contrast between 1 h and 3 h after radioligand administration.

## 3. Discussion

The tumor microenvironment or cancer stroma constitutes the critical physical part of tumors. It also promotes cancer progression by providing essential components, such as growth factors, inflammatory enzymes, cytokines, complex chemokines, and different components of the extracellular matrix [1,2,3]. The tumor microenvironment interacts with the cell signaling mechanisms and the extracellular matrix, regulating processes of immunosuppression, angiogenesis, resistance to chemotherapy, tumor growth, and metastasis. CAFs, with high expression of FAP, play a fundamental role in dynamic communication networks within the tumor microenvironment. In fact, CAFs induce a cancerous phenotype and are responsible for the production of proteolytic enzymes, paracrine growth factors, and extracellular matrix components [2]. Therefore, the development of radioligands that target FAP/CAFs in the tumor microenvironment is useful for disease diagnosis, prognosis, and therapeutic management of various cancers. 

The radioligands directed at FAP, currently used in clinical studies, are based on the N-(4-quinolinoyl)-glycyl-(2-cyano-4,4-difluoropyrrolidine) framework for labeling with ^68^Ga (e.g., ^68^Ga-FAPI-04), ^18^F (FAPI-74), ^99m^Tc (FAPI-34), and ^64^Cu, as well as therapeutic radionuclides such as ^90^Y, ^177^Lu, ^188^Re, and ^225^Ac [7,8,9,10,11,12,13]. [^99m^Tc]Tc-iFAP, evaluated in this research, is a novel SPECT radioligand based on the proline-boronic acid structure, which is coupled to the active center of FAP with high affinity (*Ki* = 0.54 nM) via Ser-624, Phe-351, Glu-203, Phe-350, and Glu-204 residues [19]. 

The biokinetics of [^99m^Tc]Tc-iFAP in humans were comparable with those reported for [^68^Ga]Ga-FAPI-04, [^18^F]F-FAPI-74, and [^99m^Tc]Tc-FAPI-34, regarding fast whole-body elimination [5,6,11,19]. In particular, [^99m^Tc]Tc-FAPI-34 has been used in patients for the diagnosis of ovarian and pancreatic cancer, demonstrating a rapid tumor uptake and fast clearance from the body. Although both [^99m^Tc]Tc-FAPI-34 and [^99m^Tc]Tc-iFAP show adequate and equivalent tumor contrasts, [^99m^Tc]Tc-FAPI-34 is prepared by using ^99m^Tc-tricarbonyl ([^99m^Tc(CO)_3_]^+^) technology, which involves several labeling steps and a post-labeling purification process due to the low radiochemical yield [11]. In contrast, [^99m^Tc]Tc-iFAP is obtained in a single labeling step from lyophilized formulations with high radiochemical purities (>98%) due to the use of the HYNIC technology [19].

An adequate concentration of the [^99m^Tc]Tc-iFAP radioligand in the primary tumors and lymph node metastases of the cases included in this study was observed, as well as concordance with the lesions detected via [^18^F]FDG PET/CT. The dosimetric results and findings described in the T/B ratios of the primary and metastatic lesions suggest that [^99m^Tc]Tc-iFAP imaging is a safe and potentially useful tool to assess FAP expression in the tumor microenvironment of various solid tumors, such as breast, lung, and cervical cancer. However, a wide application is expected in the imaging of desmoplastic tumors with high FAP expression—especially in those not detectable by ^18^F-FDG PET, which include some types of metastatic colorectal tumors (peritoneal carcinomatosis) and hepatic, duodenal, and gastric cancer [15,16,17]. 

The significant difference in the T/B ratios among the different types of cancer and between primary tumors and lymph node metastases found in this research justifies further clinical studies to possibly establish a relationship between [^99m^Tc]Tc-iFAP tumor uptake and the cancerous phenotype related to the disease prognosis, in order to support therapeutic management. However, the presence of FAP in tumors must be corroborated by immunohistopathology or PCR.

On the other hand, the coupling of DOTA-iFAP (1,4,7,10-tetraazacyclododecane-N′,N″,N‴-tetraacetic-iFAP) to the surface of [^177^Lu]Lu_2_O_3_ nanoparticles would produce a colloidal solution for theranostic purposes with high tumor retention, due to a combination of the enhanced permeability and retention effect and the active mechanism, as previously reported [23]. Another possibility for therapeutic purposes is the use of DOTA-iFAP for labeling with ^213^Bi (T_1/2_ = 45.6 min, α-emitter), under the assumption that, due to the relatively short half-life of FAP inhibitors in tumors [11,19], ^177^Lu (T_1/2_ =6.7 d, β-emitter) and ^225^Ac (T_1/2_ = 10 d, α-emitter) are not likely the most appropriate radionuclide pair for diagnostic radio-FAPIs. 

It is important to note that the relatively short tumor retention time of FAP inhibitors is not attributable to low ligand affinity. The main reason is that FAPIs target a dynamic protein expressed in cells of the tumor microenvironment, unlike other radioligands that target highly expressed proteins on the surface of cancer cells in a specific malignancy type, such as the prostate-specific membrane antigen (PSMA) and the somatostatin receptors in advanced prostate cancer and neuroendocrine tumors, respectively. For example, the tumor/background ratio for PSMA-617 averages 38 (range from 4 to 154) at 3 h [24], and up to 35 for octreotide in liver metastases [25]. Nevertheless, high FAP expression is only associated with desmoplastic tumors, where [^99m^Tc]Tc-iFAP SPECT/CT could be a useful guide for a theranostic approach to treating different types of cancer. 

## 4. Materials and Methods

### 4.1. Reagents

iFAP (((R)-1-((6-hydrazinylnicotinoyl)-D-alanyl)pyrrolidin-2-yl) boronic acid) (MW 321 g/mol) lyophilized kits were obtained from ININ (Ocoyoacac, Mexico) with GMP certification [18]. [^99m^Tc]-sodium pertechnetate was eluted from a ^99^Mo/^99m^Tc generator (GETEC, ININ, Ocoyoacac, Mexico). Most of the chemical substances were acquired from Millipore (Burlington, MA, USA).

### 4.2. [^99m^Tc]Tc-iFAP Preparation

The iFAP freeze-dried kit was reconstituted with a [^99m^Tc]TcO_4_Na/0.2 M phosphate buffer (1:1 *v/v*, 2 mL, 740 MBq) solution, and after incubation in a block heater (92 °C, 15 min), the radiochemical purity (R.P.) of the [^99m^Tc]Tc-iFAP radioligand was greater than 98%, as revealed by reversed-phase HPLC analysis (Discovery C18 column, particle size of 5 µm, 2.5 cm length, 0.46 cm I.D.; Supelco, Millipore, Burlington, MA, USA) applying the following linear gradient: flow rate of 1 mL/min, 0.1% TFA/water (A) (from 100 to 50%, over 10 min, maintained for 10 min, 30% over 5 min, and returned to 100% over 5 min), 0.1% TFA/acetonitrile (B). 

ITLC-SG (Instant thin layer chromatography-silica gel) strips (Pall Corporation, Port Washington, NY, USA) were also used for the evaluation of the [^99m^Tc]Tc-iFAP R.P. The mobile phases were the following: (1) 2-butanone (used for the identification of free [^99m^Tc]TcO_4_Na (*Rf* = 1), where [^99m^Tc]Tc-iFAP, the radiocolloid, and [^99m^Tc]Tc-EDDA/tricine remained in the origin (*Rf* = 0)), (2) sodium citrate (0.1 M, pH 5), which identified free [^99m^Tc]TcO_4_Na (*Rf* = 1) and [^99m^Tc]Tc-EDDA/tricine(Rf = 1), while [^99m^Tc]Tc-iFAP and the radiocolloid remained in the origin (*Rf* = 0), and (3) methanol-ammonium acetate (1:1 *v/v*), used as the phase for the determination of the radiocolloid (*Rf* = 0) (*Rf* = 1 for [^99m^Tc]TcO_4_Na, [^99m^Tc]Tc-iFAP, and [^99m^Tc]Tc-EDDA/tricine).

### 4.3. Volunteers and Patients

A total of 6 healthy volunteers (age range: 27–31 years; mean ± SD age, 30 ± 1.8 years; 1 female and 5 males) were included. Subjects with clinical disease or surgery were not considered for the study. Medical examination and medical background were accomplished. The mean (± SD) weight of the healthy subjects was 64.6 ± 10 kg (range: 52–77 kg). After receiving full information about the study objectives, all volunteers signed a consent form. Healthy subjects received an activity of 740 MBq (50 µg of iFAP peptide).

Three cancer patients participated in the study (2 women and 1 man; age range: 51–70 years; mean age ± SD: 62.3 ± 8.2 years), diagnosed with breast, lung, and cervical cancer (Table 3), who had a history of [^18^F]FDG PET/CT prior to the acquisition of images with [^99m^Tc]Tc-iFAP, with an interval of one day. Both the volunteers and the patients had breakfast 2 h before the [^99m^Tc]Tc-iFAP study. In addition, fasting was indicated to the patients before the scanning with [^18^F]FDG. Oncological diagnoses were corroborated histopathologically. 

This study was performed at the Department of Nuclear Medicine of the National Institute of Cancerology (INCan, Mexico City, Mexico). The patients gave informed consent. The Institutional Ethics Committee approved the protocol based on the institutional ethical standards related to human experimentation, the Declaration of Helsinki, and the basis of microdose studies. 

### 4.4. Image Acquisition

[^99m^Tc]Tc-iFAP SPECT images in healthy subjects were obtained with a SPECT/CT Symbia TruePoint camera (Siemens, Munich, Germany), with low-energy and high-resolution collimators. The parameters were adjusted as follows: matrix size: 256 × 1024 pixels; speed: 12 cm/min; window: at 140 keV (symmetric at 20%); dispersion corrections: dual energy window method (20% width at 119 keV). For correction of body attenuation, the transmission factors were estimated utilizing the I/I_0_ ratio of the count, with (I) and without (I_0_) the patient, from a flood source filled with [^99m^Tc]TcO_4_Na (740 MBq). Whole-body scintigraphy (anterior and posterior) was obtained at 0.5, 2, 4, and 24 h after i.v. application of the [^99m^Tc]Tc-iFAP radioligand.

In patients, SPECT/CT images of the affected anatomical region (chest in breast and lung cancer patients; abdomen/pelvis in cervical cancer patient) were acquired 3 h after administration of [^99m^Tc]Tc-iFAP (740 MBq) using a window centered at 140 keV with dispersion correction, a 128 × 128 array, 90 images of 8 s, and 360-degree rotation. Low-dose CT parameters were used to obtain the attenuation correction map. Reconstruction of the raw data was carried out using a Butterworth filter (cutoff: 0.5; 5th order) and an iterative procedure of ordered sets and subsets (8 iterations/4 subsets).

[^18^F]FDG imaging of patients was carried out using a PET/CT scanner (Excel 20, Siemens, Munich, Germany). The helical computed tomography parameters were a slice thickness of 5 mm, 180 mAs, and 120 kVp. After intravenous injection of [^18^F]FDG, whole-body scans were obtained at 1 h, and at 2–3 min per bed position in 3D mode from the vertex to mid-thigh. A 2D expectation maximization algorithm was used for PET image reconstruction. 

### 4.5. Image Analysis

Images obtained with [^99m^Tc]Tc-iFAP and [^18^F]FDG were examined on a Siemens VG60 multimodal workstation. Visual and semi-quantitative analyses were carried out by two nuclear medicine physicians with >9 years of experience using the volumetric analysis software.

Tumor/background ratios (T/B) were calculated by delineating volumetric regions of interest (VOIs) (3D) around tumor areas (T) and healthy tissue as background (B), including four sites: mediastinum (T/Bm), liver (T/Bl), psoas muscle (T/Bp), and contralateral healthy tissue (T/Bc). In the [^99m^Tc]Tc-iFAP images, the activity (Bq/cm^3^) was quantified (number of total counts corrected for attenuation, background, and dispersion, according to the calibration standard), and in the [^18^F]FDG images, the maximum standardized uptake value (SUVmax) was quantified.

### 4.6. Biokinetics and Dosimetry

The activity in source organs was expressed as a fraction of the whole-body activity calculated from the images of healthy subjects (ImageJ software). [^99m^Tc]Tc-iFAP biokinetics in the blood were calculated from activity–time data in the heart content. The ICRP 89 reference blood masses for adult males (5600 g) and females (4100 g) were used to scale the [^99m^Tc]Tc-iFAP activity concentration [26].

A 2D-planar/3D-SPECT hybrid activity quantitation method was used to fit the biokinetic models as previously reported [27]. In brief, planar and SPECT/CT images were obtained at 0.5 h after [^99m^Tc]Tc-iFAP administration, with a dual-head gamma camera (SPECT/CT). Correction factors ($CF_{h}$) between images were calculated by dividing the activity in the source organs quantified by SPECT (reconstructions in kBq/cm^3^) by the activity measured on planar images. Subsequently, planar images were obtained at 2, 4, and 24 h. $CF_{h}$ obtained for the source organs were used in the quantitation of $A{\left(t\right)}_{P}$ (planar process) to establish the $A{\left(t\right)}_{VOI}\;$(volumetric activity) fitted to exponential models, as follows (Equation (2)):

(2) $$A{\left(t\right)}_{VOI}=CF_{h}\times A{\left(t\right)}_{P}$$

The total nuclear transformations (N) that occurred in the source organs were calculated from the mathematical integration of $A{\left(t\right)}_{VOI}$ (Equation (3)):

(3) $$N=\int_{0}^{\infty }A{\left(t\right)}_{VOI}dt$$

The OLINDA/EXM software was used to determine the radiation doses by entering the N values to be processed [27]. The ICRP 30 GI tract model was used, assuming 0.008–0.014 as the activity fraction excreted to the small intestine (1.1 ± 0.3% of the injected activity entering the small intestine, calculated from the images as described below) [28].

### 4.7. Tumor Tissue Samples

All patients underwent biopsy of the primary tumor lesion. The histopathological studies and reports were provided by the INCan’s Pathology Department, which were interpreted by a certified and experienced pathologist.

### 4.8. Statistical Analysis

Continuous variables were summarized through mean values and relative standard deviations (SDs). Differences among tumor/background (T/B) ratios, calculated from the images of [^99m^Tc]Tc-iFAP SPECT/CT and [^18^F]FDG PET/CT patients, were analyzed by three-way ANOVA applying Tukey’s multiple comparisons test (alpha of 0.05). 

## 5. Conclusions

An adequate concentration of the [^99m^Tc]Tc-iFAP radioligand in the primary tumors and lymph node metastases of patients with breast, cervical, and lung cancer was achieved. [^99m^Tc]Tc-iFAP SPECT/CT was useful to obtain primary and metastatic lesion images of different tumors, which correlated with those detected by [^18^F]FDG PET/CT. The dosimetric results and findings described in the T/B ratios of the tumors suggest that [^99m^Tc]Tc-iFAP imaging is a safe and potentially useful tool to assess FAP expression in the tumor microenvironment. The results obtained in this research validate the performance of additional clinical studies to determine the utility of [^99m^Tc]Tc iFAP in the diagnosis and prognosis of different types of solid tumors.

## Figures and Tables

**Table 1:** Mean biokinetic model of [^99m^Tc]Tc-iFAP and total nuclear transformations (N) (MBq^.^h/MBq) (mean ± SD) in source organs calculated from six healthy volunteers using a hybrid 2D-planar/3D-SPECT methodology.

Organ	Biokinetic Model $A{\left(t\right)}_{VOI}$	$N=\overset{t=\infty }{\underset{t=0}{\int }}A{\left(t\right)}_{VOI}dt$
Liver	$A{\left(t\right)}_{VOI}=5.55e^{-21.315t}+3.38e^{-0.33t}+0.234e^{-0.139t}$ *R*^2^ = 0.99	1.11 × 10^−1^ ± 1.74 × 10^−2^
Kidneys	$A{\left(t\right)}_{VOI}=5.55e^{-21.215t}+3.52e^{-0.445t}+0.535e^{-0.166t}$ *R*^2^ = 1	1.22 × 10^−1^ ± 7.69 × 10^−2^
Urinary bladder	$A{\left(t\right)}_{VOI}=37.6\;e^{-3.245t}+8.98e^{-0.245t}+1.68e^{-0.245t}$ *R*^2^ = 0.99	5.02 × 10^−1^ ± 1.23 × 10^−1^
Remainder of the body	$A{\left(t\right)}_{VOI}=5.55e^{-21.215t}+96.4e^{-0.348t}+8.35e^{-0.384t}$ *R*^2^ = 0.99	3.04 ± 0.37

*VOI* = volume of interest.

**Table 2:** Equivalent and effective doses of [^99m^Tc]Tc-iFAP obtained from six healthy subjects (1 female and 5 males).

Target Organ	Equivalent Doses (mSv/MBq) (Mean ± SD)
Adrenals	(2.23 ± 0.35) × 10^−3^
Brain	(1.36 ± 0.21) × 10^−3^
Breast	(1.14 ± 0.18) × 10^−3^
Gallbladder Wall	(2.30 ± 0.36) × 10^−3^
LLI Wall	(2.99 ± 0.45) × 10^−3^
Small Intestine	(2.48 ± 0.37) × 10^−3^
Stomach Wall	(1.93 ± 0.30) × 10^−3^
ULI Wall	(2.31 ± 0.36) × 10^−3^
Heart Wall	(1.87 ± 0.29) × 10^−3^
Kidneys	(7.01 ± 1.09) × 10^−3^
Liver	(2.23 ± 0.35) × 10^−3^
Lungs	(1.63 ± 0.26) × 10^−3^
Muscle	(1.74 ± 0.27) × 10^−3^
Ovaries	(3.02 ± 0.47) × 10^−3^
Pancreas	(2.27 ± 0.35) × 10^−3^
Red Marrow	(1.73 ± 0.29) × 10^−3^
Skin	(1.11 ± 0.17) × 10^−3^
Spleen	(2.02 ± 0.32) × 10^−3^
Testes	(2.10 ± 0.33) × 10^−3^
Thymus	(1.62 ± 0.25) × 10^−3^
Thyroid	(1.64 ± 0.25) × 10^−3^
Urinary Bladder Wall	(2.15 ± 0.34) × 10^−3^
Uterus	(4.27 ± 0.67) × 10^−3^
Effective Dose (mSv/MBq)	(3.12 ± 0.49) × 10^−3^

**Table 3:** Patient characteristics before [^99m^Tc]Tc-iFAP and [^18^F]FDG imaging. Oncological diagnoses were confirmed via histopathological studies.

No.	Age (Years)	Gender	Clinical Status	Type of Cancer	Extent of Cancer
1	70	Female	Initial assessment	Breast cancer (ductal carcinoma; SBR 8, G3, moderate DR, Ki67 70%). Triple-negative.	Primary, lymph node
2	51	Male	Initial assessment	Lung cancer, NSCLC (adenocarcinoma).	Primary, lymph node
3	66	Female	Recurrence	Cervical cancer (squamous cell carcinoma).	Lymph node

DR: desmoplastic reaction; NSCLC: non-small cell lung cancer; SBR: Scarff–Bloom–Richardson grading; Ki67: cell proliferation index.

**Table 4:** Tumor/background (T/B) ratios evaluated with the [^99m^Tc]Tc-iFAP and [^18^F]FDG radiotracers in cancer patients.

Primary Tumors					
Type of Cancer (No. of Patients)	Tracer	T/Bm	T/Bl	T/Bp	T/Bc
Breast (1)	[^99m^Tc]Tc-iFAP	3.7	3.3	4.1	3.1
	[^18^F]FDG	4.2	2.9	7.4	8.3
Lung (2)	[^99m^Tc]Tc-iFAP	8.8	5.3	10.2	7.6
	[^18^F]FDG	4.4	2.9	8.1	10.9
Cervical (3)	[^99m^Tc]Tc-iFAP	n.a.	n.a.	n.a.	n.a.
	[^18^F]FDG	n.a.	n.a.	n.a.	n.a.
Average (1–3)	[^99m^Tc]Tc-iFAP	6.3 ± 3.6	4.3 ± 1.4	7.2 ± 4.3	5.4 ± 3.2
	[^18^F]FDG	4.3 ± 0.1	2.9 ± 0.0	7.8 ± 0.5	9.6 ± 1.8
**Lymph Node Metastases**					
Breast (1)	[^99m^Tc]Tc-iFAP	2.2	2.0	2.5	1.9
	[^18^F]FDG	2.8	2.0	5.0	5.7
Lung (2)	[^99m^Tc]Tc-iFAP	2.2	1.3	2.6	1.9
	[^18^F]FDG	3.6	2.4	6.7	9.1
Cervical (3)	[^99m^Tc]Tc-iFAP	8.3	2.7	7.8	8.4
	[^18^F]FDG	4.2	2.1	7.2	6.3
Average (1–3)	[^99m^Tc]Tc-iFAP	4.2 ± 3.5	2.0 ± 0.7	4.3 ± 3.0	4.1 ± 3.8
	[^18^F]FDG	3.5 ± 0.7	2.2 ± 0.2	6.3 ± 1.2	7.0 ± 1.8

T/Bm: tumor/mediastinum; T/Bl: tumor/liver; T/Bp: tumor/psoas muscle; T/Bc: tumor/contralateral healthy tissue; n.a.: not applicable because no recurrent primary lesion was observed at the pelvic level.

## Data Availability

Data are contained within the article.
